# Species Sorting of Benthic Invertebrates in a Salinity Gradient – Importance of Dispersal Limitation

**DOI:** 10.1371/journal.pone.0168908

**Published:** 2016-12-22

**Authors:** Alf B. Josefson

**Affiliations:** Department of Bioscience, Aarhus University, Roskilde, Denmark; University of Waikato, NEW ZEALAND

## Abstract

The relative importance of environment and dispersal related processes for community assembly has attracted great interest over recent decades, but few empirical studies from the marine/estuarine realm have examined the possible effects of these two types of factors in the same system. Importance of these processes was investigated in a hypothetical metacommunity of benthic invertebrates in 16 micro-tidal estuaries connected to the same open sea area. The estuaries differed in size and connectivity to the open sea and represented a salinity gradient across the estuaries. The Elements of Metacommunity Structure (EMS) approach on estuary scale was complemented with a mechanistic variance partitioning approach on sample scale to disentangle effects of factors affecting assembly of three trait groups of species with different dispersivity. A quasi-Clementsian pattern was observed for all three traits, a likely response to some latent gradient. The primary axis in the pattern was most strongly related to gradients in estuary salinity and estuary entrance width and correlation with richness indicated nestedness only in the matrix of the most dispersive trait group. In the variance partitioning approach measures of turnover and nestedness between paired samples each from different estuaries were related to environmental distance in different gradients. Distance between estuaries was unimportant suggesting importance of factors characterizing the estuaries. While the high dispersive species mainly were sorted in the salinity gradient, apparently according to their tolerance ranges towards salinity, the two less dispersive traits were additionally affected by estuary entrance width and possibly also area. The results exemplify a mechanism of community assembly in the marine realm where the niche factor salinity in conjunction with differential dispersal structure invertebrates in a metacommunity of connected estuaries, and support the idea that dispersive species are more controlled by the environment than less dispersive species.

## Introduction

Finding biological explanations of beta diversity, the change of diversity, is a way to identify the factors underlying biodiversity [1]. Based on niche theory, beta diversity was earlier assumed to be primarily determined by environmental variation between local communities *e*.*g*. [2]. More recently, a growing body of theoretical and observational work suggests that community assembly is additionally driven by stochastic processes such as immigration and extinction and therefore the ability of individuals to disperse among local communities may be an important determinant of species richness at all scales [3–5]. If communities were purely dispersal assembled one would expect a high unpredictability in species composition among sites in an otherwise homogeneous environment, while niche assembled communities are expected to have more predictable composition at sites in the same environment [6]. However, the usefulness of this dichotomy has been questioned recently [7, 6, 8]. In nature, community assembly may result from several interacting factors like species—environment relationships together with stochastic dispersal and demographic processes [9]. Chase [6] suggested from experiments that the relative importance of dispersal and niche processes will depend on the harshness of the environmental filter, and the interaction between the environment and dispersal may also be influenced by species functions or ecological traits [7, 10]. For instance, differences in dispersivity traits were of major importance for community assembly in metacommunities [11, 12], which by definition are communities connected by dispersal [4]. Specifically, Cottenie [11] found that a major determinant of the relative importance of local environmental (niche) and neutral (dispersal) processes was whether or not species exhibited passive dispersal or some more active dispersal mode, the latter including pelagic larvae dispersal, and stressed that sufficient dispersal is needed for environmental processes to effectively structure communities [13]. Therefore species sorting in disturbance gradients may be most obvious for dispersive species [14, 15], not only because dispersal limitation is less likely and they quickly can occupy more places than less dispersive species [16] but also because dispersive species may be better to track environmental differences [17]. When niche differences are due to different tolerance levels towards some environmental factor, sorting of species may sometimes result in nested patterns [9, 18, 19].

There are, however, few field studies addressing these issues in the marine/estuarine environments [20], but see [21], and particularly the effects of both environmental and dispersal related factors in the same system. Previous cross system studies of estuaries with different connectivity to the adjacent sea have mostly focused on alpha, being the net result of assembly processes, and conclusions have either emphasized importance of internal environmental factors for assembly [22–24] or dispersal limitation [25]. Assembly processes like turnover were not specifically addressed in these studies and it was not clear how much of the different richness in the estuaries was due to dispersal from the adjacent sea and how much was due to environmental sorting of species inside the estuaries.

Here, I investigate the possible importance of niche related and dispersal related factors for community assembly in a hypothetical metacommunity, *sensu* [4] consisting of benthic invertebrate assemblages in 16 estuaries connected by the same sea area in the Baltic Sea—North Sea transition. The fauna of each estuary was defined as a local community. Since dispersal was one key interest the fauna was partitioned into three trait groups of species with different reproductive modes, hypothetically translated into different dispersivity. The estuaries have different entrance widths, potentially influencing connectivity with the adjacent open sea, and at the same time they represent a salinity gradient from ca. 28 to 10 psu. The system likely differs functionally from other marine metacommunities, where local communities are embedded in gradients and the main dispersal barrier is the geographical distance between them [26]. In the present metacommunity the environmental gradient is represented by the different levels of the environmental factor in the areas of each local community, which are connected to each other via a large assumedly reasonably homogeneous open sea species pool. It assumed that the major dispersal regulating factor for these communities is the entrance width of the estuaries which regulates salt water flow and likely propagule input into (and out of) the estuaries. The levels of the environmental factor salinity in the estuaries then eliminate invaders that cannot tolerate the local environment and thereby causes sorting of species among the estuaries. So, while in an open sea metacommunity some sorting may occur already before invaders reach the local community, main sorting in the present metacommunity will occur in the local community (estuary).

I use a dual analytical approach on the same dataset at two different spatial scales to identify structuring factors in the metacommunity with separate analyses on the three different dispersive trait groups of species, which together made up the total number of species in the system. First, I use the Elements of Metacommunity Structure (EMS) approach [27–29] on estuary level to identify the distribution pattern along the main latent gradient. The second approach was applied on sample level, and environmental distance in latent gradient variables are directly correlated with measures of turnover and nestedness between estuaries. The rationale for an additional approach to EMS was the difficulty in this approach to separate factors affecting the axes of variation in the RA analysis, and to detect effects of dispersal [30]. I specifically address the following hypotheses:

1. Dispersal trait identity is important for local community assembly in the estuaries
2. Dispersive species are relatively more controlled by the estuarine environment than less dispersive species, which in turn are relatively more controlled by dispersal limitation or spatial factors like fragment area.

The hypotheses were corroborated by the results.

## Materials and Methods

### Study area—predictor variables

**Fig 1:**
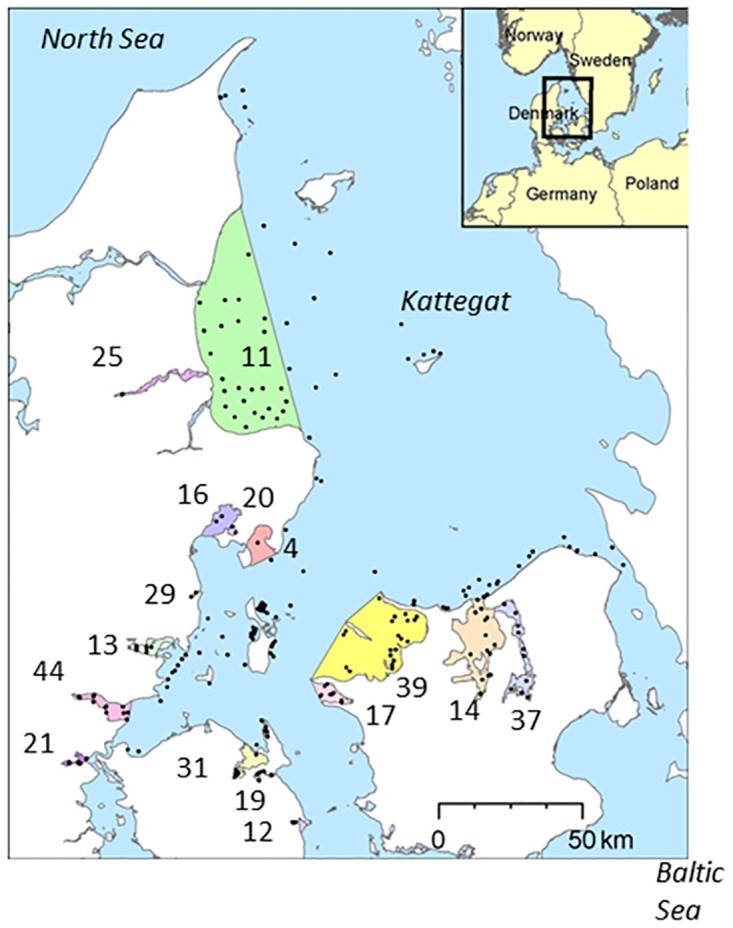
Map over the investigation area showing locations of 16 estuaries with areas indicated by different colors and id numbers as in Table 1. Open sea is indicated by blue color and the 242 sampling sites by black dots. Copyright on the base map by the Danish Geodata Agency.

The studied system contained a series of 16 estuaries fringing ca. half of the coastline of a marine sea area with sampling sites at similar water depths as in the estuaries (<15 m), and with a hypothetically large species pool (Table 1, Fig 1).

**Table 1:** Environmental variables and sampling data for 16 micro-tidal Danish estuaries.

Estuary	Estuary id nr	Area (km^2^)	Entrance width (km)	Average estuary salinity	Average salinity outside entrance	Nr of sites	Nr of samples
Holckenhavn Fjord	12	0.5	0.22	9.5	18	3	10
Norsminde Fjord	29	1.7	0.20	11.5	27	2	12
Knebel Vig	20	7.7	0.83	23	27	2	2
Kerteminde Fjord	19	8.7	0.04	17.5	20	6	6
Kolding Fjord	21	14.9	1.51	18	21	7	58
Horsens Fjord	13	45.7	2.22	22.5	22.5	7	64
Mariager Fjord	25	45.7	0.67	17	28	1	37
Kalundborg Fjord	17	56.5	9.60	17.5	21	11	11
Odense Fjord	31	60.8	0.46	17.5	22.2	6	29
Vejle Fjord	44	73.5	6.65	20	22.5	12	31
Kalø Vig	16	77.9	4.10	27	27	2	24
Ebeltoft Vig	4	84.3	11.15	27.6	27.6	1	3
Roskilde Fjord	37	124.2	0.86	15	22.5	10	42
Isefjord	14	315.6	4.78	18.5	22.5	16	50
Sejero Bugt	39	674.2	39.39	23.2	23.2	22	22
Aalborg og Hevring Bugt	11	1683.5	80.08	27.9	27.9	27	104

Salinity values represent conditions in bottom water at water depths of fauna sampling sites. Each sample covers a bottom area of ca. 0.1 m^2^.

Predictor variables:

1. **Estuary area**. Large areas often contain more species than small areas. Since the studied estuaries differ widely in area, estuary area was included as a predictor.
2. **Estuary entrance width**. The estuaries have entrance widths differing over 3 orders of magnitude (Table 1), and since the entrance width was a main predictor of salt water flushing from the sea [31] with potential influence on transport of pelagic larvae, post-settling juveniles and even some adults, the estuary openings may represent a gradient in strength of connectivity between the estuaries and the sea.
3. **Aquatic distance** between the mid points of the entrances of the estuaries was used as predictor of change in beta diversity with distance between estuaries. As a consequence of distance decay in similarity beta may increase with distance [32].
4. **Average salinity** at benthic sites in the estuaries. The relative importance of salt water flushing from the sea and the diffuse freshwater inputs differs among the estuaries and therefore the set of estuaries also represented a gradient in terms of salinity (Table 1, and well documented in [33, 25]), a key factor affecting species richness in the present area [34–36]. The salinity gradient across estuaries was equally strong as the open sea gradient from the North Sea/Kattegat to the south-western Baltic Sea (30–10 psu, [36,37]). Because the freshwater inputs to the selected estuaries are mainly diffuse, and at least not concentrated to major rivers in the heads, the salinity gradients in the estuaries are relatively weak. This is indicated by the fact that for 12 of the 16 estuaries, the difference between average salinity at benthic fauna site depths outside the estuary entrance and in the estuaries were less than 4 psu and for 5 of these the average salinity was at the same level (Table 1).

Predictors 1, 2 and 4 were used in the EMS analysis and all 4 predictors in the variance partitioning analysis.

### Invertebrate fauna sampling

Fauna data is at present stored in the Environmental Database ODAM (maintained by the Danish Centre for Environment and Energy, Aarhus University, Denmark). Samples were collected either with a Van Veen grab (covering 0.1 m^2^ bottom area) or in most cases the smaller Haps sampler (covering ca. 0.013 m^2^ bottom area), a box-core type of sampler. In the estuaries 19% of the samples were collected with a Van Veen grab. To obtain the same sampled area the smaller samples from each sampling occasion were pooled to samples with ca. 0.1 m^2^ bottom area. Since the pooled samples captured some beta diversity between subsamples, the richness was on average higher, ca. 33%, than in the non-pooled samples in the estuaries. However, a Wald-Wolfowitz Runs test on the ratios of pooled samples/total samples from each estuary versus the main gradient of salinity did not show a significant serial trend (P>0.05, cut point 0.5, n = 16), and regressions based on each type of sample showed similar trends of alpha over the gradient, suggesting no bias in relation to the major trend in the system. A varying number of replicate 0.1 m^2^ samples were available from the estuaries (Table 1). All samples came from un-vegetated sedimentary bottoms (sand-silt-mud). A total of 778 samples for fauna were taken at 242 sites with varying frequency in the time period 1990–2007 (Fig 1, Table 1). Of these, 505 samples were from 135 sites in estuaries and 273 from 107 sites in the open sea pool area (Fig 1), positioned in the same water depth interval as in the estuaries *i*.*e*. <ca.15 m (S3 File). The fauna was extracted from the sediments using standard methods such as extraction with 1 mm sieve [38], and determined to lowest possible taxon (mostly species), and counted.

### Defining dispersivity traits

The invertebrate species/taxa were categorized into groups of species with different reproductive traits, excluding the very few true freshwater species. It was assumed that this categorization related to different dispersivity [37] (S1 File). The species were grouped into those with planktotrophic larval development *i*.*e*. high dispersive species with small feeding larvae with long pelagic life, in the following abbreviated HD, and species with direct benthic development, being low dispersive, in the following abbreviated LD. While the last mentioned group will entirely depend on post-settlement transport of juveniles or adults, the first mentioned group will have the options of both pelagic larvae dispersal and post-settlement dispersal. Categorization into these two groups was fairly certain and hypothetically represented two extremes in terms of dispersal distance potential. In the remaining group of species most species has some pelagic larvae phase, many with large non-feeding lecithotrophic larvae often with shorter pelagic life than those of the planktotrophic species. Therefore this group, in the following abbreviated ID group, was assumed to have intermediate dispersal distance potential.

### Alpha

*Alpha* species richness at both the sample scale and the estuary scale was the number of taxa including species and some higher taxa like genera found in a sample covering ca. 0.1 m^2^ bottom area and an estuary respectively (S1 File).

### Elements of Metacommunity Structure analysis (EMS)

The EMS approach uses a stepwise procedure and can simultaneously test for multiple idealized patterns across a set of sites [27]. In contrast to ordering sites along a specific environmental variable, the EMS analysis allows the metacommunity itself to define the gradient of response. First, the site-by-species incidence matrices, where sites are estuaries, were ordered with reciprocal averaging (RA). Then objective criteria based on coherence, turnover, and boundary clumping were used to assess the correspondence of the empirical data set with each of the hypothetical idealizations of species distribution (*i*.*e*., checkerboard, nested, evenly spaced, Gleasonian, or Clementsian patterns) [27,28,26].

The significance of the index value for coherence and turnover was tested using a fixed-proportional null model, which maintains species richness of each site (*i*.*e*., row sums are fixed), but species ranges are filled based on their marginal probabilities (*i*.*e*., the “r1” null model, [39, 40]. I used 1000 simulations to provide random matrices, with zero rows allowed in the null matrices. Index values derived from randomization were then compared to the observed index values to assess statistical significance. I interpreted the results of the EMS analysis according to [28], and used the metacommunity function in the “metacom” package for calculations and the Image function for graphs of sorted matrices, ([39], in the R environment, R Core Team R version 3.3.1, 2016-06-21). The data used for the EMS analysis were presence of taxa determined to species and in a very few cases higher taxa where there was reason to assume that it represented only one and the same species (S2 File). Spearman rank correlation was used to test whether latent environmental gradients (*i*.*e*., primary axis site scores from RA in correspondence analysis) were significantly correlated with measured predictor variables (*i*.*e*. salinity, entrance width and estuary area), as well as with species richness [28, 30].

It is known since long that the marine species in the study area have different tolerance ranges towards salinity [41, 42], with several ranges reaching from fully marine (>30) in the Kattegat to < 5 in the Baltic Sea. The distribution of ranges are often thought to overlap so that “most marine and estuarine organisms can withstand full sea water, but some of them cannot withstand lowered salinities and thus the species numbers decline with the salinity gradient decline in estuaries.” [43]. Because the estuaries have environments with different salinity regimes, I predict a metacommunity structure with species sorted among the estuaries according to their tolerance ranges in the salinity gradient, and because of considerable overlaps between many ranges I expect some nestedness. Since sufficient dispersal is needed for effective sorting in environmental gradients [44–46], I predict the clearest pattern for the most dispersive species.

### Variance partitioning analysis

Response variables were measures of *Beta* diversity between pairs of samples based on presence-absence with each sample from different estuaries:

The Sørensen dissimilarity index (β_sor_)

$$\beta \text{sor}=\frac{\text{b}+\text{c}}{2\text{a}+\text{b}+\text{c}}$$ (1)

Where a = the number of species in common of two samples, b = species number unique to one sample and c = species number unique to the other sample.

Two additive components of β_sor_, (β_sim_ and β_nes_), were calculated in order to separate variation in species composition due to replacement and variation due to nested patterns [47, 1, 48]:

Replacement beta (β_sim_)

$$\beta \text{sim}=\frac{\text{min}\left(\text{b},\text{c}\right)}{\text{a }+\text{ min }\left(\text{b},\text{c}\right)}$$ (2)

Nested beta (β_nes_)

$$\beta \text{nes }=\;\beta \text{sor }–\;\beta \text{sim}=\frac{│b\;-\;c\;│}{2\text{a}+\text{b}+\text{c}}\times \frac{\text{a}}{\text{a}+\text{min}\left(\text{b},\text{c}\right)}$$ (3)

The Sørensen index and its additive components were computed in the R statistical language (R Development Core Team 2009) using the “betapart” package ver.1.3, 2013-12-12 by A. Baselga, D. Orme, S. Villeger, J. De Bortoli and F. Leprieur.

Relationships between predictor variables and pair-wise beta where analyzed by distance based linear modelling using the model option DistLM in PERMANOVA+ for PRIMER [49], where each beta value was the average value of all between-estuary comparisons of samples from two estuaries.This method uses permutation tests, which is appropriate for the present material which includes similarity matrices as response variables. Since there were more than one predictor variable both marginal and forward selection sequential estimation were used to partition the variances. Marginal estimation gives the explained proportions of the variance (R^2^) when predictor variables are fitted alone while forward selection sequential estimation gives the explained contributions to total variance after the previous predictor variable (-s) has been fitted. All P-values were determined from 999 permutations.

It is predicted that turnover between two samples will increase, and nestedness will decrease with increasing environmental distance in the gradient of importance. This is because in a sorted pattern where tolerance ranges shift along a gradient it is likely that species composition in samples far away in the gradient are more different, *i*.*e*. turnover is higher and nestedness lower, than between close samples in the gradient.

## Results

### Species distributions overview

A total of 515 species/taxa and 285,487 individuals were found in the 778 samples from the 242 sites in the investigation area. Of these, 92 species occurred only in estuaries and 132 only in the open sea (Table 2). Thus 57% of the species were found both in estuarine and open sea environments. The two extremes of dispersivity, HD and LD traits, had overall similar species numbers, but in the former group far more species occurred both in estuarine and open sea environments (74%) than in the LD species group (44%). The corresponding figure for the ID trait group was 54%.

**Table 2:** Species/taxa number overview of dispersive traits and all species in the estuaries and the open sea.

Trait	Total area	In estuaries only	In open sea only	Percentage in common estuary-open sea
**High dispersive (HD)**	134	13	22	73.9
**Intermediate dispersive (ID)**	258	50	70	53.5
**Low dispersive (LD)**	123	29	40	43.9
**All species**	515	92	132	56.5

Dispersive traits equates different reproductive traits, where HD = pelagic planktotrophic larval development, LD = direct benthic development, often brooding, and ID = remaining species most with lecithotrophic larval development and short pelagic stage.

### EMS analysis

**Fig 2:**
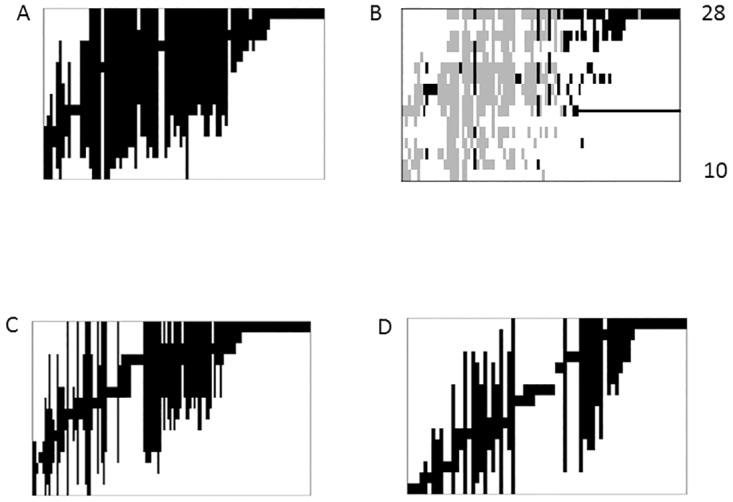
Sorted site-by-species incidence matrices for invertebrate species in 16 estuaries, with species on the x-axis and sites (estuaries) on the y-axis. Matrices for HD trait (A), ID trait (C) and LD trait (D) result from reciprocal averaging and show species ranges (black columns) hiding embedded absences. Matrix B shows the incidence matrix for the HD trait with the species ordered as in matrix A and with sites (estuaries) ordered after falling salinity from the top and with gradient end values indicated. Horizontal solid line indicates the 18 psu limit. Species recorded from the low saline SW Baltic Sea, south of the Danish Straits, are indicated by grey boxes. Black boxes indicate species never recorded south of the Danish straits *i*.*e*. at salinities < ca. 18 psu. Matrices with estuary id and species names are given in S2 File.

Ordinated site-by-species matrices (Fig 2) of all three traits had fewer embedded absences than predicted from the null model and showed highly significant coherence (Table 3). The turnover, *i*.*e*. the number of replacements were higher than expected from the null model, but not significant (P>0.05), for all three traits. Clumping was highly significant for all three traits with high values > 1 of the Morisita’s index. A coherent metacommunity with non-significant positive turnover and positive boundary clumping has quasi-Clementsian structure [28] indicating response to an underlying environmental gradient across estuaries of groups of species with similar distributions [28]. In such a pattern species ranges contain fewer embedded absences and species tend to replace each other more often than expected from null model simulations.

**Table 3:** Results of analysis of coherence, species turnover and boundary clumping for invertebrates in 16 estuaries.

	Coherence				Species turnover				Boundary clumping		
	Number of absences	P	Mean	SD	Number of replacements	P	Mean	SD	Morisita’s index	P	df
HD	423	<0.001	743	7.21	13888	0.114	9012	55.58	6.720	<0.001	90
ID	722	<0.001	1231	9.00	33652	0.647	28825	102.66	8.369	<0.001	135
LD	368	<0.001	522	6.58	16496	0.531	13797	65.62	3.908	<0.001	67

df = no. of species -3, HD = High dispersive, ID = Intermediate dispersive and LD = Low dispersive trait.

### Predictor variables and richness associated with structuring gradient

I use the non-parametric Spearman’s r to investigate the associations between the site scores on the primary axis obtained from reciprocal averaging and a subset of environmental variables, including estuary salinity, entrance with and area (Table 4). The highest correlation for each trait group were between site scores and salinity (P<0.001 for HD and LD and P<0.01 for ID), followed by entrance width (P<0.001 for HD, P<0.01 for LD and P<0.05 for ID). However, the three environmental factors were inter-correlated with high rank concordance between them with a Spearman’s r of 0.78 (P<0.01) for area vs entrance width, 0.75 (P<0.01) for entrance width vs salinity and 0.58 (P<0.05) for salinity vs area, n = 16). Correlation of richness measures with site scores showed a highly significant result (P<0.01) only for the HD trait indicating nestedness in the matrix of this group (*e*.*g*. [30]).

**Table 4:** Spearman’s correlation coefficients between site (estuary) scores on the primary axis from RA and predictor variables and richness.

	Sal	Entrance	Area	Spec/estuary	Nr taxa/estuary
HD trait	0.906***	0.865***	0.590**	0.694**	0.620 **
ID trait	0.631**	0.497*	0.149 ns	0.406 ns	0.337 ns
LD trait	0.794***	0.774**	0.528*	-0.019 ns	-0.058 ns

Sal = Estuary salinity, Entrance = Estuary entrance width, Area = Estuary area, Spec/estuary = Species richness of estuary, No taxa/estuary = Taxa richness of estuary, n = 16,

*** = P<0.001,

** = P<0.01,

* = P<0.05,

ns = P > 0.05.

### Variance partitioning analysis of Beta between estuaries

**Fig 3:**
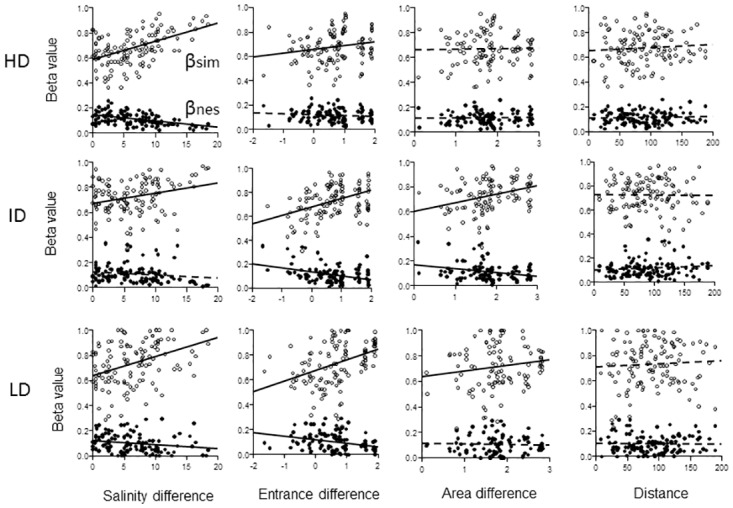
Plots of β_sim_ and β_nes_ for each trait group (panel rows) vs 4 predictors (panel columns). Solid regression lines indicate significant effect (P<0.05) and broken line no effect (P>0.05) of the predictor using marginal tests. HD = High dispersive, ID = Intermediate dispersive and LD = Low dispersive trait groups. Salinity difference = Absolute difference between estuaries of average estuary salinity, Entrance difference = Log10 of Absolute difference in estuary entrance width (km), Area difference = Log10 of Absolute difference between estuary areas (km^2^), Distance = the shortest water way distance (km) between the centers of the estuary entrances.

**Fig 4:**
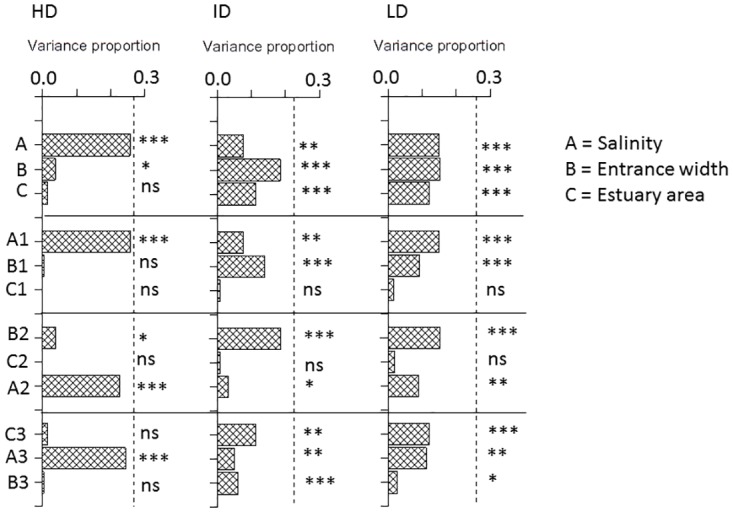
Results of marginal and sequential tests in DistLM modeling of β_sim_ with three predictors (A = Salinity difference, B = Log10 Entrance width difference and C = Log10 Estuary area difference) of paired samples from different estuaries. HD = High dispersive, ID = Intermediate dispersive and LD = Low dispersive species trait group. Variance proportion = the proportion of total variance explained by the predictor (S^2^). Top sequence (A,B,C) shows results from marginal tests and the following three sequences (A1,B1,C1 and B2,C2,A2 and C3,A3,B3) show results from sequential tests, where A1, B2 and C3 are first fitted to data and B1,C1,C2,A2, A3, B3 gives the remaining independent variance explained after the previous variable has been fitted. Dashed vertical line indicates the total variance proportion explained by the variables. *** = P = 0.001, ** = P <0.01, * = P < 0.05 and ns = P > 0.05.

**Fig 5:**
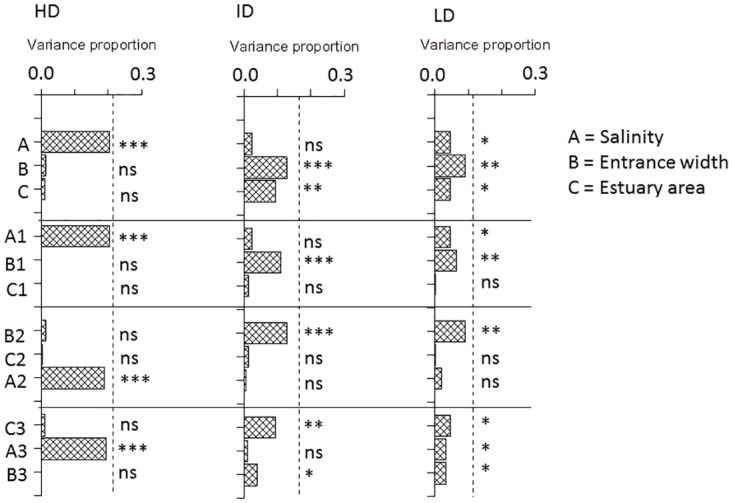
Results of marginal and sequential tests in DistLM modeling of β_nes_ with three predictors (A = Salinity difference, B = Log10 Entrance width difference and C = Log10 Estuary area difference) on paired samples from different estuaries. For more information see legend of Fig 4.

Matrices of Sørensen beta (β_sor_) and its two additive components replacement beta (β_sim_) and nested beta (β_nes_) for each of the three dispersive trait groups of species were related to matrices of distance between estuaries, and between-estuary differences of salinity, estuary entrance width and estuary area (Fig 3). There were no effects what-so-ever of the spatial variable inter-estuary distance on any of the beta measures (Fig 3, Table 5). The results of the DistLM analysis, marginal and sequential tests, with the remaining three predictors are summarized in Fig 4 for β_sim_ and in Fig 5 for β_nes_. Results for β_sor_ were similar to results for β_sim_ and therefore given in S1 Fig.

**Table 5:** Results of marginal tests in DistLM modeling of the pair-wise beta measures βsim, βnes and βsor versus 4 predictor variables.

Trait	Predictor	βsim			βnes			βsor			df
		R^2^	F	P	R^2^	F	P	R^2^	F	P	
**HD**											
	Salinity	0.257	40.87	**0.001**	0.204	30.29	**0.001**	0.208	30.97	**0.001**	118
	Distance	0.0064	0.755	0.389	0.0063	0.753	0.378	0.0225	2.72	0.093	118
	Entrance	0.0372	4.56	**0.040**	0.014	1.68	0.204	0.0405	4.98	**0.031**	118
	Area	0.0155	1.86	0.190	0.0112	1.333	0.254	0.0132	1.576	0.205	118
	All predictors	0.268			0.214			0.241			
**ID**											
	Salinity	0.0758	9.68	**0.003**	0.0236	2.853	0.102	0.105	13.85	**0.002**	118
	Distance	0.0004	0.043	0.850	0.0133	1.592	0.222	0.0051	0.598	0.435	118
	Entrance	0.185	26.748	**0.001**	0.128	17.307	**0.001**	0.161	22.57	**0.001**	118
	Area	0.113	15.1	**0.001**	0.0945	12.31	**0.002**	0.0839	10.81	**0.002**	118
	All predictors	0.224			0.164			0.221			
**LD**											
	Salinity	0.150	20.78	**0.001**	0.0471	5.837	**0.017**	0.201	29.62	**0.001**	118
	Distance	0.0035	0.408	0.512	0.00001	0.0015	0.958	0.0074	0.88	0.376	118
	Entrance	0.152	21.2	**0.001**	0.0911	11.83	**0.002**	0.161	22.59	**0.001**	118
	Area	0.121	16.25	**0.001**	0.0464	5.735	**0.020**	0.152	21.21	**0.001**	118
	All predictors	0.260			0.114			0.323			

Test results are given for each of the three dispersive trait groups: HD = High dispersive, ID = Intermediate dispersive and LD = Low dispersive trait groups. S^2^ = the explained proportion of total variance, F = Pseudo-F, Salinity = Absolute difference between estuaries of average estuary salinity, Entrance = Log10 of Absolute difference in estuary entrance width (km), Distance = the shortest water way distance (km) between the centers of the estuary mouths, Area = Log10 of Absolute difference in estuary area (km^2^). df = number of comparisons -2, where each comparison is the average beta value of all between estuary pairwise comparisons of samples in two estuaries. Results are from 999 permutations. Significant P-values (P<0.05) are in bold.

For the HD group there was a highly significant and largely independent effect (P = 0.001), on all beta measures only of salinity difference and the independent variance explained by this predictor accounted for most of total variance explained by all (three) predictors (Figs 4 and 5, sequences 2 and 3 in left panel). There was a small effect of entrance width difference (P<0.05, marginal) on β_sim_ (and β_sor_), but the variance explained by this predictor overlapped almost completely with the variance explained by salinity (Fig 4, sequence 1in left panel). Salinity difference was the only significant predictor of β_nes_ (P = 0.001) accounting for nearly all of total explained variance (Fig 5, left panel, Table 5).

Results for the ID and LD groups were fairly similar. There were small but significant effects on β_sim_ (P<0.05 and P<0.01) of salinity difference independent of the variances explained by the other predictors (Fig 4, sequence 2 middle and right panels), but no independent effects on β_nes_ (Fig 5). There were highly significant marginal effects (P<0.001) of both entrance width difference and area difference on β_sim_ with the greatest effect of entrance width, but the variances explained by area overlapped completely with the variance explained by entrance width, which explained a significant independent part (P = 0.001 for ID and P<0.05 for LD) of total variance after the other predictors had been fitted (Fig 4, sequence 3 middle and right panels). The results for β_nes_ of these two groups were similar, but less significant than for β_sim_ (Fig 5, P<0.05 for both groups) and the results for β_sor_ were nearly identical with β_sim_ (S1 Fig). Directions of significant trends in beta measures were similar among predictors with increase of β_sim_ and decrease of β_nes_ with increasing difference in in predictor value (Fig 3).

### Beta between estuaries and open sea

**Fig 6:**
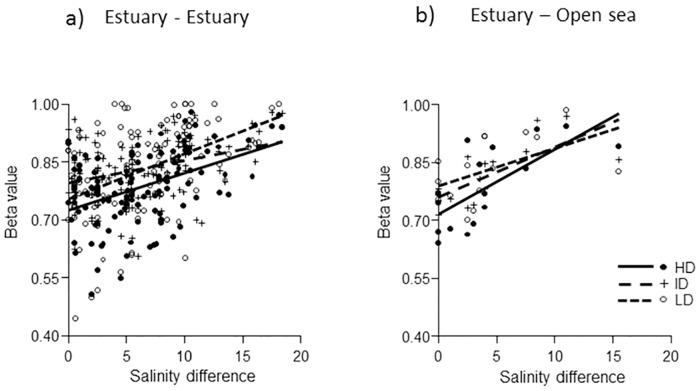
Plots of β_sor_ vs salinity difference of the three dispersal groups. a): Beta vs differences between estuaries and b): Beta vs differences between estuaries and adjacent open sea.

Salinity difference between estuaries was a main predictor significantly affecting β_sor_ between samples from different estuaries of all three trait groups (S1 Fig), showing an increasing beta with increasing difference (Fig 6a). The average β_sor_ between samples from each estuary and samples in the adjacent open sea area was also positively related to difference between estuaries and open sea salinity (Fig 6b) and showed fairly similar regressions for all three traits. All regressions were significant at P<0.05 (OLR, n = 16).

### Alpha in the estuaries

**Fig 7:**
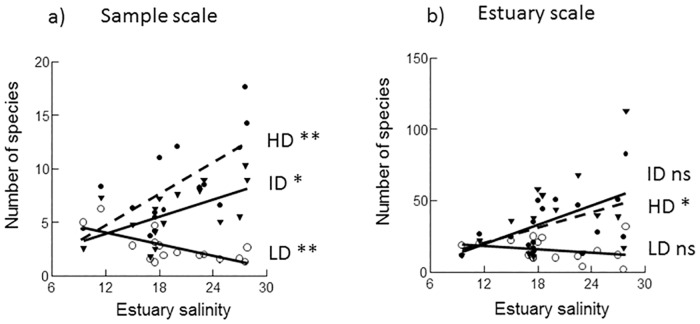
Alpha of dispersal trait groups at two spatial scales plotted against average estuary salinity. a): Sample scale and b): Estuary scale. Regression lines are from ordinary linear regression (OLR, n = 16). ** = P<0.01, * = P<0.05, ns = P>0.05).

Average estuary alpha on the sample scale and total alpha on estuary scale (number of taxa per estuary) of the three trait categories were plotted and regressed (OLR, n = 16) against estuary average salinity (Fig 7). The trends in alpha across estuaries at both spatial scales of the three trait groups differed between traits but were fairly similar between scales. The HD group showed significant (P<0.05) increases of alpha at both scales with the steepest increase at sample scale (P<0.01). Alpha of the ID group increased significantly (P<0.05) at the sample scale and close to significant (P<0.10) at estuary scale, while the LD group decreased significantly (P<0.01) at the sample scale and was unchanged (P>0.05) at estuary scale (Fig 7). Trends and significance levels in number of species per estuary, used in the ÉMS analyses, were similar to number of taxa with, as expected, somewhat lower intercepts (not shown for brevity).

## Discussion

### Translating reproduction mode into dispersivity traits

The rationale for the translation was based on both biological properties of the reproduction modes, where much information on individual species was obtained from the seminal work of Thorson [50] and empirical studies indicating different occupancy patterns for different reproductive groups.

Defining dispersivity as the likelihood of establishing at a site with increasing distance from the parents it is both a function of the ability of the larvae (propagules) to be transported far away and the number of successfully settling larvae (*i*.*e*. propagule pressure). Some recent studies *e*.*g*. [12] suggested that the efficiency of dispersal for passive dispersers, like invertebrates with pelagic larvae, decreased with increasing propagule size. The high dispersive trait group (HD) included only species with planktotrophic larvae development which have small feeding larvae with long pelagic life and thus potential for long distance dispersal. Moreover planktotrophs often produce many larvae per adult which give potential for a high density of larvae. The intermediate dispersive group (ID) comprised species with lecithotrophic larvae development with non-feeding larvae mostly larger than the feeding larvae and with shorter pelagic life than the HD species, although short duration of pelagic life has been questioned for some of these species [51]. The low dispersive group (LD), dominated by *Peracarida* crustaceans, has direct benthic development and is solely dependent on post-settlement dispersal *i*.*e*. of juveniles or adults. While earlier marine studies often have equated high dispersal with pelagic larval life, some recent studies have emphasized importance of post-settlement dispersal for community assembly *e*.*g*. [52]. However, since this type of dispersal may be used by many species in all three groups, and the options of pelagic larvae dispersal are only available for the HD and ID groups, it seems reasonable to assume a higher dispersivity of these groups than of the LD group.

Empirical studies in the North-Eastern Atlantic area [53, 37] showed that marine benthic invertebrate species with planktotrophic larvae development had higher occupancy than species with direct benthic development, in agreement with the idea that planktrophs were more dispersive than species with direct benthic development. Dispersive species often have lower levels of beta diversity and lower rates of distance decay in similarity between sites [32] than non-dispersive species [54, 1, 55]. In the open Kattegat planktotrophic species had lower Sørensen beta diversity (β_sor_) between sites and lower rate of distance decay *sensu* [32] than species with direct benthic development at the scale of tens of km. This indicated that dispersal limitation matters for community assembly of species with direct benthic development in these open areas with high connectivity among sites due to water currents [37].

Some observations in the present study gives further circumstantial evidence of high dispersivity of planktotrophic (HD) species: 1) HD species have the largest populations indicated by on average the highest number of individuals in the samples. The average number of individuals per sample in the estuaries of HD species was ca. 300 (286), for ID species 70 and for LD species 43 individuals. 2) A much higher proportion (74%) of HD species occurred in both estuarine and open sea environments compared to the LD species (44%, Table 2). 3) Furthermore, the HD species had a much higher alpha compared to the other groups at the local sample scale than at the scale of estuaries and sea area (Fig 7). For instance, the ratio of average alpha of HD versus LD species was 3.8 in samples from all estuaries compared to 1.4 for total species numbers. On average ca. half of total alpha (51%) at the sample scale was due to HD species compared to 29% of total species numbers in the estuaries. This is also in line with the view that dispersal capability increases diversity at the local scale relative to the regional scale [56].

### Elements of Metacommunity Structure, EMS

Species distribution patterns across the estuaries were coherent for all three dispersive traits indicating response to some latent environmental gradient (Fig 2). This together with the subsequent analyses of turnover and clumping in the EMS approach indicated a quasi-Clementsian structure [28] where partly overlapping distribution ranges of species shifted along this gradient indicating species sorting in the gradient. Such a pattern has recently been reported for invertebrates in other estuarine environments [26]. It cannot be ruled out, however, that some of the highly significant clumping is due to some truncation of the salinity gradient (see [28]) as the full gradient in the area, relevant for species with marine affinity, goes from > 30 to ca. 5 psu. Although the primary axis of each trait group was correlated with all three gradient factors which all were inter-correlated, the highest Spearman’s r was with salinity for all traits (Table 4). The regression analysis between the primary axis and species richness indicated nestedness in the site-by-species matrix of HD species, but nestedness as such was not directly related to structuring factors, nor was turnover, the opposite of nestedness. The HD group had the pattern most similar to Clementsian with P = 0.11 for turnover (Table 3). To check that this pattern resulted from sorting in a salinity gradient, geographical distributions in the Baltic Sea—North Sea/Kattegat area was examined for individual species of the HD group (S4 File). Species were categorized into those recorded south of the Danish Straits in the western Baltic Sea at salinities < ca. 18 psu, and those species that never (seldom) were recorded south of the Danish Straits. Panel B in Fig 2 shows that most species in latter category occurred only in the upper part of the gradient, while the species in the former category occurred in the lower part of the gradient and in many cases were distributed over a wide range along the gradient. It appears thus, that the “estuary samples” of the open sea species pools are non-random, and reinforce the idea that sorting of this group has occurred according to tolerance ranges towards salinity.

### Variance partitioning analysis of Beta between estuaries

The results from the DistLM analysis indicated that factors characterizing the estuaries were important for assembly, while the spatial factor distance between estuaries had no effect at all. This suggests that dispersal limitation due to geographical distance between estuaries was less important for assembly than other factors. This was not unexpected given that the open sea species pool outside the estuaries was reasonably homogeneous and the pool area having only a weak salinity gradient from N to S. From a pattern, such as the one detected by the EMS analysis (Fig 2), I expected an increase in turnover and a decrease in nestedness with increasing environmental distance between samples along the gradient. Results further suggested different reasons for change in turnover and nestedness in different dispersive groups. As pointed out by Carvalho *et al*. [57], β_sim_ is the proportion of species in the poorer sample which is not nested into the richer sample (Eq 2), and consequently β_sim_ is not only a measure of replacement but also an inverse measure of nestedness (1- β_sim_) between samples, *i*.*e*. the proportion of the poorer sample that is nested into the richer sample. At the same time β_nes_, being the heterogeneity due to nested patterns [47, 1, 48], is positively influenced by the proportion of species in the poorer sample being nested into the richer sample (a/ (a + min (b, c)) in Eq 3). It is therefore likely that the trends with increasing distance of gradient factors (Fig 3) *i*.*e*. an increase in β_sim_ and decrease of β_nes_ both reflects a decrease in nestedness in the gradient. The significant pattern in the present study was nearly identical with a simulated pattern of the two beta measures where richness differences were constant and “a, the number of species in common” decreased in a gradient [57].

Beta measures of the HD group were strongly, and only, affected by the absolute difference in estuary salinity. The smooth increase in β_sim_, thus reflecting at the same time increasing turnover and decreasing nestedness between samples, and decrease in β_nes_ with increasing difference in salinity likely resulted from sorting according to different tolerance ranges in the salinity gradient. Beta of ID and LD species were also affected by salinity difference but less significant than beta of HD. While HD species were largely unaffected by the connectivity factor (entrance width) and estuary area (Table 5, Fig 3), both beta measures of the ID and LD groups were highly significantly (P = 0.001 mostly) affected by these two factors with marginal tests. However effects of these two predictors cannot only partly be disentangled as shown by the sequential DistLM tests where the explained variances of area difference overlap completely with the variance explained by connectivity (Figs 4 and 5). Connectivity explained significantly more (P<0.01 for ID and P<0.05 for LD) of the total variance than area (Figs 4 and 5). In summary, the gradient analysis on sample scale indicated significant effects on HD trait species only of salinity, while there were independent effects on the ID and LD groups of both salinity and estuary entrance, where turnover increased and nestedness decreased with increasing environmental distance in the gradients.

Salinity in these micro-tidal areas may be viewed as a niche variable which creates an environmental filter across estuaries, potentially generating nested patterns along the gradient. The form of the pattern may vary depending on the overlap structure of tolerance ranges and the degree of dispersal limitation of the potential colonizing species. The estuary entrance width is a variable acting before the environmental sorting inside the estuary and may cause some sorting according to different dispersal ability and also rareness. Spreading of pelagic larvae as well as post-settling stages is likely directional following the water currents and in the present case the salt water currents (in and out) between the open sea and the estuaries. Since estuary entrance width regulates salt water inflow to the estuaries [31] it is a potential measure of connectivity with, or isolation from, the adjacent open sea. It seems intuitively reasonable to expect that a wide entrance will allow larvae of more different species to enter the estuary than a narrow entrance width. At the same time it may be assumed that the entering success will depend on the density of larvae. While species with many larvae during long time periods, like the HD species, may be able to enter the estuary already with narrow entrance widths, species with few larvae or juveniles during short periods, like many species in the ID and LD groups may need wider entrance widths to enable entering. This could be one explanation as to why beta of HD species are unaffected by entrance width differences, whereas ID and LD groups are affected.

### Nestedness and alpha

Nestedness was indicated by the EMS analysis in the site-by-species matrix of HD species, and nestedness decreased with increasing environmental distance in the gradients in the variance partitioning analysis. A nested pattern arises when smaller communities contain significant subsets of the species in larger communities through ordered species loss or gain [58]. There may be several reasons for geographical nestedness and a common one may be habitat fragmentation which may generate nested patterns if fragments differ in size and relative isolation [59]. A mechanism could then be that smaller fragments selectively lose specialist species with low abundance and if fragments are isolated recolonization is difficult for these species [60]. In this study estuaries may be viewed as fragments with widely different sizes (areas) and with different isolation due to different entrance widths. Nested patterns may also occur in environmental gradients [61, 9] if there are ordered sequences of absences along the gradient due to different environment requirements/tolerance towards the gradient factor. Here, such a pattern appears in the salinity gradient, most clear for HD species (Fig 2), where distribution ranges overlap and particularly the lower range limits varies with salinity. A consequence of such a nested pattern is likely an increase in alpha with increasing salinity, and indeed this seems to be the case for HD species (Fig 7). Since in the EMS analysis nestedness was indicated only for HD species, where both richness and salinity were strongly correlated with the primary axis in the pattern and the decrease in nestedness with environmental distance in the salinity gradient was strongest (most significant) for HD species (Table 5), the nested pattern likely explains the significant increase in alpha of this group at both estuary (P<0.05) and sample (P<0.01) scales (Fig 7). Alpha of the two less dispersive groups, ID and LD, showed diverging trends which were only significant at sample scale (P<0.05 and P<0.01 respectively, Fig 7) and these groups were affected by other factors in addition to salinity.

### Complementarity of approaches

While in the past research has either focused on the pattern-based approaches like EMS or on mechanistic approaches with little coupling between mechanism and structure [28]. More recently research combining these types of approaches have often proven useful to get insight into community assembly [30] (and references therein). In this study I use the two different statistical approaches, one pattern-based and one mechanistic, on the same data set to infer structuring factors in a metacommunity at two different spatial resolutions [30]. While the EMS approach identified the main distributional pattern along the main environmental gradient (s), the variance partitioning at sample scale identified independent effects of salinity on assembly of HD species and of salinity and dispersal on assembly of the two less dispersive traits. Thus, the findings at the sample scale complement the results from the EMS analyses at estuary scale and imply that the reasons for the quasi-Clementsian pattern at estuary scale, to some extent differed between dispersive trait groups. This together with the findings of different trends of alpha across the estuaries ordered after salinity provides support for both hypotheses (1–2)

1. Dispersal trait identity is important for local community assembly in the estuaries.
  This is in line with studies in other systems showing that reproductive traits together with other traits like body size may be useful when unravelling assembly mechanisms in meta-communities [11, 12].
2. Dispersive species are relatively more controlled by the estuarine environment than less dispersive species, which in turn are relatively more controlled by dispersal limitation or spatial factors like fragment area.
  This is in line with studies in freshwater systems [55, 14] finding that high dispersive species were more under environmental control and less affected by spatial processes than low dispersive species.

So, the present results indicate that assembly in these estuaries results from sorting inside the estuaries and dispersal from the outside sea area, and is probably the first example of an estuarine/marine metacommunity with effects of dispersal on environmental sorting.

## Conclusions

- EMS analysis identified a quasi-Clementsian structure of the metacommunity with estuaries as local communities where the main axis from correspondence analysis was significantly correlated with gradients in salinity, entrance width and area, all inter-correlated. This suggested species sorting in some latent environmental gradient.
- Different alpha and beta patterns for groups of species with different reproductive modes functionally translated into different dispersivity, indicate that dispersal trait identity is important for community assembly of benthic invertebrates in some Danish estuaries.
- Species in the most dispersive species group are sorted only in the salinity gradient across estuaries likely according to tolerance towards salinity, while the two less dispersive trait groups were affected by both salinity and dispersal limitation due to restricted connectivity to the sea, possibly together with estuary size.
- The results exemplify a mechanism of community assembly in the marine realm where a niche factor act in conjunction with dispersal and support the hypothesis that dispersive species is more under environmental control than less dispersive species.
- The main dispersal limiting factor in this metacommunity is estuary entrance width which is effective at the proximity of the local community and thereby differs from many other published metacommunities in several realms where geographical distance between local communities limits dispersal.

## Supporting Information

S1 File Datasets with species abundance in samples from all sampling sites. Dataset A: High dispersive (HD) species with planktotrophic larvae development. Dataset B: Intermediate dispersive (ID) species mostly with lecithotrophic larvae development. Dataset C: Low dispersive (LD) species with direct benthic development, often brooding. Positions and water depths of sites are given in S3 File.
(XLSX)

S2 File Sorted matrices of occurrence (presence) in the 16 estuaries with species names and estuary id. Matrix A: High dispersive (HD) species with planktotrophic larvae development. Matrix B: Intermediate dispersive (ID) species mostly with lecithotrophic larvae development. Matrix C: Low dispersive (LD) species with direct benthic development, often brooding. Filled box denotes presence and unfilled denotes absence. Columns representing estuaries are ordered after falling estuary salinity from left to right. Rows representing species are sorted after falling row sums (richness) from top to bottom. In the matrix of HD species (Matrix A) grey boxes indicate species recorded from the low saline SW Baltic Sea, South of the Danish Straits, and black boxes indicate species never recorded south of the Danish Straits *i*.*e*. at salinities < ca. 18 psu.
(XLSX)

S3 File List of sampling sites with positions, water depths, and bottom water salinity. (DOCX)

S4 File Literature references on geographical distribution of species. (DOCX)

S1 Fig Results of marginal and sequential tests in DistLM modeling of β_sor_ with three predictors (A = Salinity difference, B = Log10 Entrance width difference and C = Log10 Estuary area difference) of paired samples from different estuaries. HD = High dispersive /planktotrophic species, ID = Intermediate dispersive /Lecithotrophic species and LD = Low dispersive / direct benthic development species group. Variance proportion = the proportion of total variance explained by the predictor (S^2^). Top sequence (A,B,C) shows results from marginal tests and the following three sequences (A1,B1,C1 and B2,C2,A2 and C3,A3,B3) show results from sequential tests, where A1, B2 and C3 are first fitted to data and B1,C1,C2,A2, A3, B3 give the remaining independent variance explained after the previous variable has been fitted. Dashed vertical line indicates the total variance proportion explained by the variables. *** = P = 0.001, ** = P <0.01, * = P <0.05 and ns = P >0.05.
(TIF)
