# Supplementary material for: Species Sorting of Benthic Invertebrates in a Salinity Gradient – Importance of Dispersal Limitation
Source: PLoS One. 2016 Dec 22;11(12):e0168908. doi: 10.1371/journal.pone.0168908 (PMC5179068; doi:10.1371/journal.pone.0168908)
Supplement: S3 File — (DOCX) [file pone.0168908.s003.docx]

List of stations with positions, water depths and bottom water salinity.

| Estuary, Sea area | Longitude E (Decimal degrees) | Latitude N (Decimal degrees) | Water depth (m) | Salinity | Estuary id | Station id |
| --- | --- | --- | --- | --- | --- | --- |
| Ebeltoft Vig | 10.598500 | 56.174233 | 15.0 | 27.60 | 4 | s9 |
| Aalborg og Hevring Bugt | 10.482033 | 56.567567 | 8.1 | 27.87 | 11 | s43 |
| Aalborg og Hevring Bugt | 10.677867 | 56.591650 | 4.2 | 27.87 | 11 | s44 |
| Aalborg og Hevring Bugt | 10.416133 | 56.687783 | 4.4 | 27.87 | 11 | s45 |
| Aalborg og Hevring Bugt | 10.640367 | 56.562733 | 8.0 | 27.87 | 11 | s46 |
| Aalborg og Hevring Bugt | 10.537000 | 56.617733 | 12.2 | 27.87 | 11 | s47 |
| Aalborg og Hevring Bugt | 10.429867 | 56.601233 | 7.8 | 27.87 | 11 | s48 |
| Aalborg og Hevring Bugt | 10.482033 | 56.567567 | 7.6 | 27.87 | 11 | s49 |
| Aalborg og Hevring Bugt | 10.595867 | 56.578567 | 7.8 | 27.87 | 11 | s50 |
| Aalborg og Hevring Bugt | 10.545700 | 56.535733 | 7.7 | 27.87 | 11 | s51 |
| Aalborg og Hevring Bugt | 10.723033 | 56.559400 | 10.3 | 27.87 | 11 | s52 |
| Aalborg og Hevring Bugt | 10.757167 | 56.582167 | 6.4 | 27.87 | 11 | s53 |
| Aalborg og Hevring Bugt | 10.584833 | 56.652333 | 14.2 | 27.87 | 11 | s54 |
| Aalborg og Hevring Bugt | 10.644700 | 56.655233 | 7.8 | 27.87 | 11 | s55 |
| Aalborg og Hevring Bugt | 10.751367 | 56.653067 | 10.2 | 27.87 | 11 | s56 |
| Aalborg og Hevring Bugt | 10.777200 | 56.612400 | 8.0 | 27.87 | 11 | s57 |
| Aalborg og Hevring Bugt | 10.427217 | 56.649400 | 6.8 | 27.87 | 11 | s58 |
| Aalborg og Hevring Bugt | 10.511700 | 56.658067 | 8.1 | 27.87 | 11 | s59 |
| Aalborg og Hevring Bugt | 10.521500 | 56.933333 | 9.7 | 27.87 | 11 | s60 |
| Aalborg og Hevring Bugt | 10.661700 | 56.832467 | 11.0 | 27.87 | 11 | s61 |
| Aalborg og Hevring Bugt | 10.353300 | 56.767733 | 5.0 | 27.87 | 11 | s62 |
| Aalborg og Hevring Bugt | 10.297167 | 56.926683 | 4.2 | 27.87 | 11 | s63 |
| Aalborg og Hevring Bugt | 10.440167 | 56.933333 | 6.8 | 27.87 | 11 | s64 |
| Aalborg og Hevring Bugt | 10.583167 | 57.073833 | 7.5 | 27.90 | 11 | s65 |
| Aalborg og Hevring Bugt | 10.317333 | 56.839500 | 5.0 | 27.90 | 11 | s66 |
| Aalborg og Hevring Bugt | 10.416833 | 56.853333 | 7.0 | 27.90 | 11 | s67 |
| Aalborg og Hevring Bugt | 10.516383 | 56.866583 | 11.0 | 27.90 | 11 | s68 |
| Aalborg og Hevring Bugt | 10.667167 | 56.873333 | 13.0 | 27.90 | 11 | s69 |
| Holckenhavn Fjord | 10.751150 | 55.296617 | 0.2 | 11.56 | 12 | s70 |
| Holchenhavn Fjord | 10.755300 | 55.296217 | 0.7 | 11.56 | 12 | s71 |
| Holchenhavn Fjord | 10.770483 | 55.297050 | 1.3 | 11.63 | 12 | s72 |
| Horsens Fjord | 9.991667 | 55.853333 | 5.0 | 22.90 | 13 | s73 |
| Horsens Fjord | 9.908333 | 55.845000 | 3.0 | 22.90 | 13 | s74 |
| Horsens Fjord | 9.908333 | 55.851667 | 3.5 | 22.90 | 13 | s75 |
| Horsens Fjord | 9.908333 | 55.858333 | 3.0 | 22.90 | 13 | s76 |
| Horsens Fjord | 9.965000 | 55.838333 | 5.0 | 22.90 | 13 | s77 |
| Horsens Fjord | 9.965000 | 55.848333 | 5.0 | 22.90 | 13 | s78 |
| Horsens Fjord | 9.965000 | 55.855000 | 4.0 | 22.90 | 13 | s79 |
| Isefjord | 11.871833 | 55.803333 | 3.1 | 18.50 | 14 | s80 |
| Isefjord | 11.857667 | 55.813833 | 2.0 | 18.50 | 14 | s81 |
| Isefjord | 11.800167 | 55.934167 | 1.8 | 18.50 | 14 | s82 |
| Isefjord | 11.872000 | 55.736333 | 2.4 | 18.50 | 14 | s83 |
| Isefjord | 11.869833 | 55.736667 | 5.5 | 18.73 | 14 | s84 |
| Isefjord | 11.811000 | 55.675833 | 4.5 | 18.93 | 14 | s85 |
| Isefjord | 11.763500 | 55.804500 | 8.6 | 18.93 | 14 | s86 |
| Isefjord | 11.900167 | 55.830000 | 8.1 | 18.93 | 14 | s87 |
| Isefjord | 11.855167 | 55.908667 | 10.9 | 18.93 | 14 | s88 |
| Isefjord | 11.833500 | 55.945333 | 8.4 | 18.93 | 14 | s89 |
| Isefjord | 11.865000 | 55.916667 | 10.0 | 18.93 | 14 | s90 |
| Isefjord | 11.823333 | 55.721667 | 7.5 | 18.93 | 14 | s91 |
| Isefjord | 11.811667 | 55.680000 | 4.6 | 18.93 | 14 | s92 |
| Isefjord | 11.858333 | 55.735000 | 6.8 | 18.93 | 14 | s93 |
| Isefjord | 11.853333 | 55.860000 | 9.6 | 19.22 | 14 | s94 |
| Isefjord | 11.815667 | 55.972833 | 3.9 | 18.57 | 14 | s95 |
| Kalo Vig | 10.398733 | 56.257733 | 12.0 | 27.00 | 16 | s112 |
| Kalo Vig | 10.368733 | 56.242733 | 13.2 | 27.10 | 16 | s114 |
| Kalundborg Fjord | 10.987667 | 55.691833 | 0.0 | 19.15 | 17 | s115 |
| Kalundborg Fjord | 11.041500 | 55.672833 | 13.0 | 19.15 | 17 | s116 |
| Kalundborg Fjord | 11.040167 | 55.669500 | 11.0 | 19.15 | 17 | s117 |
| Kalundborg Fjord | 11.040167 | 55.667000 | 11.0 | 19.15 | 17 | s118 |
| Kalundborg Fjord | 10.996833 | 55.696500 | 15.0 | 19.15 | 17 | s119 |
| Kalundborg Fjord | 10.977167 | 55.693167 | 15.0 | 19.15 | 17 | s120 |
| Kalundborg Fjord | 10.963500 | 55.726167 | 6.0 | 19.15 | 17 | s121 |
| Kalundborg Fjord | 10.928833 | 55.691500 | 10.0 | 19.15 | 17 | s122 |
| Kalundborg Fjord | 10.927833 | 55.691167 | 9.0 | 19.15 | 17 | s123 |
| Kalundborg Fjord | 10.926333 | 55.688667 | 6.0 | 19.15 | 17 | s124 |
| Kalundborg Fjord | 10.951833 | 55.719500 | 15.0 | 19.15 | 17 | s125 |
| Kerteminde Fjord | 10.643100 | 55.446200 | 2.5 | 16.29 | 19 | s129 |
| Kerteminde Fjord | 10.572200 | 55.430717 | 2.0 | 16.29 | 19 | s130 |
| Kerteminde Fjord | 10.566050 | 55.448817 | 2.4 | 16.29 | 19 | s131 |
| Kerteminde Fjord | 10.600000 | 55.458333 | 5.5 | 16.88 | 19 | s132 |
| Kerteminde Fjord | 10.579833 | 55.455000 | 4.2 | 16.88 | 19 | s133 |
| Kerteminde Fjord | 10.579833 | 55.455000 | 4.2 | 16.88 | 19 | s133B |
| Knebel Vig | 10.456733 | 56.224733 | 5.8 | 23.00 | 20 | s134 |
| Knebel Vig | 10.474067 | 56.205900 | 5.0 | 23.00 | 20 | s135 |
| Kolding Fjord | 9.584333 | 55.490000 | 3.0 | 20.50 | 21 | s137 |
| Kolding Fjord | 9.530000 | 55.493333 | 4.0 | 20.50 | 21 | s138 |
| Kolding Fjord | 9.530167 | 55.496667 | 3.0 | 20.50 | 21 | s139 |
| Kolding Fjord | 9.590000 | 55.492500 | 4.5 | 20.50 | 21 | s140 |
| Kolding Fjord | 9.584333 | 55.490000 | 3.8 | 20.50 | 21 | s141 |
| Kolding Fjord | 9.578833 | 55.496667 | 4.0 | 20.50 | 21 | s142 |
| Kolding Fjord | 9.626667 | 55.506667 | 11.0 | 22.70 | 21 | s143 |
| Mariager Fjord | 9.847667 | 56.645500 | 10.0 | 15.58 | 25 | s162 |
| Norsminde Fjord | 10.219033 | 56.009567 | 1.0 | 11.50 | 29 | s172 |
| Norsminde Fjord | 10.244533 | 56.021900 | 1.0 | 11.50 | 29 | s173 |
| Odense Fjord | 10.561667 | 55.511667 | 8.5 | 22.20 | 31 | s176 |
| Odense Fjord | 10.444333 | 55.443333 | 0.5 | 22.20 | 31 | s177 |
| Odense Fjord | 10.445667 | 55.451667 | 0.7 | 22.20 | 31 | s178 |
| Odense Fjord | 10.448500 | 55.465000 | 2.0 | 22.20 | 31 | s180 |
| Odense Fjord | 10.461667 | 55.453333 | 1.0 | 22.20 | 31 | s181 |
| Odense Fjord | 10.461000 | 55.455000 | 0.5 | 22.20 | 31 | s182 |
| Roskilde Fjord | 12.075000 | 55.662500 | 4.2 | 13.30 | 37 | s200 |
| Roskilde Fjord | 12.033333 | 55.886250 | 7.6 | 13.30 | 37 | s201 |
| Roskilde Fjord | 11.963900 | 55.956817 | 3.3 | 13.30 | 37 | s202 |
| Roskilde Fjord | 12.058850 | 55.792133 | 3.9 | 13.30 | 37 | s203 |
| Roskilde Fjord | 12.066667 | 55.713000 | 4.8 | 13.30 | 37 | s204 |
| Roskilde Fjord | 12.060333 | 55.812500 | 8.0 | 13.30 | 37 | s205 |
| Roskilde Fjord | 12.025500 | 55.927833 | 7.0 | 13.30 | 37 | s206 |
| Roskilde Fjord | 12.037167 | 55.675500 | 4.3 | 13.32 | 37 | s208 |
| Roskilde Fjord | 11.981667 | 55.688333 | 4.0 | 13.32 | 37 | s209 |
| Roskilde Fjord | 12.035833 | 55.865000 | 4.5 | 17.53 | 37 | s210 |
| Sejero Bugt | 11.268500 | 55.989500 | 7.0 | 23.20 | 39 | s212 |
| Sejero Bugt | 11.460167 | 55.916167 | 7.0 | 23.20 | 39 | s213 |
| Sejero Bugt | 11.383500 | 55.858667 | 7.0 | 23.20 | 39 | s214 |
| Sejero Bugt | 11.418500 | 55.850333 | 6.0 | 23.20 | 39 | s215 |
| Sejero Bugt | 11.351000 | 55.827000 | 6.5 | 23.20 | 39 | s216 |
| Sejero Bugt | 11.326833 | 55.767833 | 7.5 | 23.20 | 39 | s217 |
| Sejero Bugt | 11.326000 | 55.779500 | 7.5 | 23.20 | 39 | s218 |
| Sejero Bugt | 11.335167 | 55.793667 | 8.0 | 24.20 | 39 | s219 |
| Sejero Bugt | 11.411000 | 55.937833 | 9.0 | 24.20 | 39 | s220 |
| Sejero Bugt | 11.320167 | 55.826167 | 9.5 | 24.20 | 39 | s221 |
| Sejero Bugt | 11.320167 | 55.810333 | 9.0 | 24.20 | 39 | s222 |
| Sejero Bugt | 11.326000 | 55.779500 | 9.0 | 24.20 | 39 | s223 |
| Sejero Bugt | 11.472667 | 55.928667 | 11.0 | 25.34 | 39 | s224 |
| Sejero Bugt | 11.423500 | 55.916167 | 11.0 | 25.34 | 39 | s225 |
| Sejero Bugt | 11.366833 | 55.868667 | 11.0 | 25.34 | 39 | s226 |
| Sejero Bugt | 11.074333 | 55.777000 | 11.0 | 25.34 | 39 | s227 |
| Sejero Bugt | 11.335167 | 55.916167 | 12.0 | 26.30 | 39 | s228 |
| Sejero Bugt | 11.075167 | 55.889500 | 12.0 | 26.30 | 39 | s229 |
| Sejero Bugt | 11.088500 | 55.762833 | 12.0 | 26.30 | 39 | s230 |
| Sejero Bugt | 11.335167 | 55.916167 | 13.0 | 27.10 | 39 | s231 |
| Sejero Bugt | 11.351000 | 55.931167 | 13.0 | 27.10 | 39 | s232 |
| Sejero Bugt | 11.062667 | 55.877833 | 15.0 | 27.60 | 39 | s233 |
| Vejle Fjord | 9.667167 | 55.683333 | 8.0 | 22.86 | 44 | s255 |
| Vejle Fjord | 9.670000 | 55.705000 | 7.0 | 22.86 | 44 | s256 |
| Vejle Fjord | 9.666833 | 55.700000 | 7.0 | 22.86 | 44 | s257 |
| Vejle Fjord | 9.828817 | 55.667367 | 4.0 | 22.90 | 44 | s258 |
| Vejle Fjord | 9.850183 | 55.627683 | 5.0 | 22.90 | 44 | s259 |
| Vejle Fjord | 9.591667 | 55.696667 | 3.5 | 22.90 | 44 | s260 |
| Vejle Fjord | 9.584333 | 55.700000 | 4.4 | 22.90 | 44 | s261 |
| Vejle Fjord | 9.833800 | 55.647583 | 13.8 | 23.80 | 44 | s262 |
| Vejle Fjord | 9.738333 | 55.670000 | 11.0 | 23.80 | 44 | s263 |
| Vejle Fjord | 9.738333 | 55.650000 | 12.0 | 23.80 | 44 | s264 |
| Vejle Fjord | 9.733667 | 55.666667 | 10.0 | 23.80 | 44 | s265 |
| Vejle Fjord | 9.856667 | 55.650000 | 13.0 | 23.80 | 44 | s266 |
| Hevring Bugt | 11.064500 | 56.691667 | 13.5 | 27.87 | Open sea | o5 |
| Hevring Bugt | 10.948167 | 56.653533 | 11.9 | 27.87 | Open sea | o12 |
| Hevring Bugt | 10.800650 | 56.713483 | 12.0 | 27.87 | Open sea | o13 |
| Aalborg Bugt | 10.950000 | 56.933333 | 13.0 | 27.90 | Open sea | o52 |
| Aalborg Bugt | 10.851667 | 57.105500 | 8.5 | 27.90 | Open sea | o53 |
| Aalborg Bugt | 11.051500 | 57.071667 | 7.5 | 27.90 | Open sea | o1 |
| Aalborg Bugt | 10.791667 | 56.856667 | 14.0 | 27.90 | Open sea | o2 |
| Anholt | 11.661500 | 56.746167 | 5.0 | 18.83 | Open sea | o95 |
| Anholt | 11.622000 | 56.755333 | 12.0 | 22.52 | Open sea | o94 |
| Anholt | 11.477367 | 56.732733 | 5.2 | 26.50 | Open sea | o14 |
| Anholt | 11.567450 | 56.744000 | 6.9 | 26.50 | Open sea | o15 |
| Anholt | 11.446183 | 56.841467 | 12.0 | 26.50 | Open sea | o16 |
| Fornes | 10.900667 | 56.497500 | 9.4 | 27.87 | Open sea | o6 |
| Kattegat, Jerup | 10.580833 | 57.534333 | 14.8 | 32.00 | Open sea | o90 |
| Kattegat, Jerup | 10.570167 | 57.587167 | 15.0 | 32.00 | Open sea | o96 |
| Kattegat, Jerup | 10.437667 | 57.568500 | 3.5 | 27.30 | Open sea | o97 |
| Kattegat, Jerup | 10.442167 | 57.567000 | 6.0 | 27.30 | Open sea | o98 |
| Kattegat, Jerup | 10.470500 | 57.573333 | 10.5 | 28.90 | Open sea | o99 |
| Læso Rende | 10.672667 | 57.163000 | 13.4 | 32.00 | Open sea | o104 |
| Fornes | 10.936450 | 56.368283 | 6.8 | 27.87 | Open sea | o3 |
| Fornes | 10.962133 | 56.359717 | 10.6 | 27.87 | Open sea | o4 |
| Fornes | 10.755533 | 56.211733 | 14.6 | 27.87 | Open sea | o17 |
| Oresund | 12.636833 | 56.060200 | 6.0 | 19.23 | Open sea | o22 |
| SjelNOkyst | 12.383000 | 56.111667 | 5.9 | 19.23 | Open sea | o24 |
| SjelNOkyst | 11.851667 | 55.982167 | 6.1 | 19.23 | Open sea | o32 |
| SjelNOkyst | 12.344000 | 56.125500 | 7.5 | 20.03 | Open sea | o25 |
| SjelNOkyst | 11.871667 | 55.989000 | 7.0 | 20.03 | Open sea | o31 |
| SjelNOkyst | 11.933833 | 56.028333 | 9.1 | 20.03 | Open sea | o108 |
| SjelNOkyst | 12.386333 | 56.113667 | 8.3 | 20.32 | Open sea | o92 |
| SjelNOkyst | 12.581667 | 56.118167 | 12.3 | 22.30 | Open sea | o40 |
| SjelNOkyst | 12.484400 | 56.098600 | 10.0 | 22.52 | Open sea | o23 |
| SjelNOkyst | 12.311967 | 56.156167 | 12.3 | 22.52 | Open sea | o26 |
| SjelNOkyst | 12.135033 | 56.116200 | 11.7 | 22.52 | Open sea | o27 |
| SjelNOkyst | 12.114500 | 56.102033 | 11.8 | 22.52 | Open sea | o28 |
| SjelNOkyst | 11.927000 | 56.020000 | 10.3 | 22.52 | Open sea | o29 |
| SjelNOkyst | 11.827933 | 56.034933 | 13.9 | 22.52 | Open sea | o30 |
| SjelNOkyst | 11.884500 | 56.016833 | 10.5 | 22.52 | Open sea | o101 |
| SjelNOkyst | 12.135500 | 56.115833 | 12.0 | 22.52 | Open sea | o118 |
| SjelNOkyst | 12.055000 | 56.073000 | 11.0 | 22.52 | Open sea | o112 |
| SjelNOkyst | 12.312500 | 56.156167 | 13.0 | 22.52 | Open sea | o91 |
| SjelNVkyst | 11.648500 | 55.951167 | 5.5 | 19.23 | Open sea | o66 |
| SjelNVkyst | 11.636833 | 55.951167 | 5.0 | 19.23 | Open sea | o67 |
| SjelNVkyst | 11.751833 | 55.987833 | 5.0 | 19.23 | Open sea | o69 |
| SjelNVkyst | 11.456833 | 55.966167 | 8.0 | 20.03 | Open sea | o65 |
| SjelNVkyst | 11.623500 | 55.954500 | 6.0 | 20.03 | Open sea | o68 |
| SjelNVkyst | 11.741000 | 56.002000 | 9.0 | 20.03 | Open sea | o70 |
| SjelNVkyst | 11.770167 | 56.012000 | 6.0 | 20.03 | Open sea | o71 |
| SjelNVkyst | 11.460167 | 55.977833 | 14.0 | 22.52 | Open sea | o72 |
| SjelNVkyst - outside | 11.248500 | 56.071167 | 13.0 | 22.52 | Open sea | o73 |
| Ebeltoft outside | 10.669017 | 56.119483 | 5.5 | 22.47 | Open sea | o7 |
| Endelave/Juelsminde Kyst | 10.075000 | 55.726667 | 15.0 | 25.70 | Open sea | o93 |
| Endelave/Julsminde Kyst | 10.181267 | 55.828117 | 5.8 | 22.90 | Open sea | o57 |
| Endelave/Julsminde Kyst | 10.160617 | 55.814500 | 7.4 | 22.90 | Open sea | o58 |
| Endelave/Julsminde Kyst | 10.136900 | 55.794517 | 12.2 | 22.90 | Open sea | o59 |
| Endelave/Julsminde Kyst | 10.107500 | 55.772267 | 13.6 | 22.90 | Open sea | o60 |
| Endelave/Julsminde Kyst | 10.082033 | 55.751683 | 3.7 | 22.90 | Open sea | o61 |
| Endelave/Julsminde Kyst | 10.121667 | 55.778333 | 14.0 | 25.20 | Open sea | o89 |
| Fynshoved | 10.594583 | 55.617183 | 0.6 | 22.20 | Open sea | o127 |
| Fynshoved | 10.623333 | 55.581833 | 1.0 | 22.20 | Open sea | o128 |
| Fynshoved | 10.624000 | 55.566667 | 1.0 | 22.20 | Open sea | o129 |
| Fynshoved | 10.616167 | 55.596333 | 2.0 | 22.20 | Open sea | o130 |
| Fynshoved | 10.613833 | 55.582667 | 1.0 | 22.20 | Open sea | o131 |
| Nordlige Lillebælt | 10.036350 | 55.684067 | 5.5 | 20.01 | Open sea | o62 |
| Nordlige Lillebælt | 9.854400 | 55.532833 | 4.7 | 20.01 | Open sea | o63 |
| Nordlige Lillebælt | 9.910500 | 55.527400 | 12.0 | 23.81 | Open sea | o125 |
| Nordlige Lillebælt | 9.910500 | 55.527400 | 12.2 | 23.81 | Open sea | o33 |
| Nordlige Lillebælt | 9.910500 | 55.527400 | 12.5 | 23.81 | Open sea | o121 |
| Nordlige Lillebælt | 9.691833 | 55.385000 | 13.0 | 23.81 | Open sea | o120 |
| Odense Fjord outside | 10.565500 | 55.541667 | 12.7 | 22.20 | Open sea | o114 |
| Samso | 10.413683 | 55.825133 | 3.2 | 21.86 | Open sea | o11 |
| Samso | Missing | Missing | 5.7 | 22.47 | Open sea | o117 |
| Samso | 10.313417 | 55.735550 | 5.1 | 22.47 | Open sea | o64 |
| Samso | 10.589917 | 55.967217 | 7.0 | 23.20 | Open sea | o166 |
| Samso | 10.605250 | 55.978383 | 7.5 | 23.20 | Open sea | o167 |
| Samso | 10.560017 | 55.872083 | 6.8 | 23.20 | Open sea | o177 |
| Samso | 10.770150 | 55.973967 | 6.5 | 23.20 | Open sea | o19 |
| Samso | 10.689900 | 55.938950 | 6.8 | 23.20 | Open sea | o20 |
| Samso | 0.000000 | 0.000000 | 9.7 | 24.20 | Open sea | o159 |
| Samso | 10.606583 | 55.973550 | 9.8 | 24.20 | Open sea | o168 |
| Samso | 10.595750 | 55.967717 | 7.8 | 24.20 | Open sea | o169 |
| Samso | 10.617750 | 55.959550 | 9.9 | 24.20 | Open sea | o175 |
| Samso | 10.560583 | 55.908050 | 10.5 | 25.34 | Open sea | o178 |
| Samso | 10.563250 | 55.897550 | 11.0 | 25.34 | Open sea | o180 |
| Samso | 10.653917 | 55.826550 | 11.7 | 25.80 | Open sea | o160 |
| Samso | 10.669250 | 55.816217 | 11.6 | 25.80 | Open sea | o161 |
| Samso | 10.626917 | 55.978383 | 11.9 | 25.80 | Open sea | o171 |
| Samso | 10.603250 | 55.962217 | 11.9 | 25.80 | Open sea | o174 |
| Samso | 10.577417 | 55.902717 | 11.9 | 25.80 | Open sea | o179 |
| Samso | 10.571750 | 55.895050 | 11.4 | 25.80 | Open sea | o181 |
| Samso | 10.564417 | 55.886883 | 11.8 | 25.80 | Open sea | o183 |
| Samso | 10.547000 | 55.880000 | 11.8 | 25.80 | Open sea | o184 |
| Samso | 10.310367 | 55.938900 | 11.9 | 25.80 | Open sea | o9 |
| Samso | 10.638167 | 55.953200 | 11.4 | 25.80 | Open sea | o185 |
| Samso | 10.661750 | 55.847717 | 12.4 | 26.30 | Open sea | o165 |
| Samso | 10.613917 | 55.984883 | 12.9 | 26.30 | Open sea | o170 |
| Samso | 10.622583 | 55.970217 | 13.0 | 26.30 | Open sea | o172 |
| Samso | 10.612917 | 55.966383 | 12.3 | 26.30 | Open sea | o173 |
| Samso | 10.638167 | 55.953200 | 12.1 | 26.30 | Open sea | o18 |
| Samso | 10.849850 | 56.079500 | 12.1 | 26.30 | Open sea | o21 |
| Samso | 10.682250 | 55.862433 | 13.9 | 27.10 | Open sea | o162 |
| Samso | 10.509917 | 55.793050 | 14.0 | 27.10 | Open sea | o176 |
| Samso | 10.549083 | 55.889383 | 13.7 | 27.10 | Open sea | o182 |
| Samso | 10.387367 | 55.881400 | 13.2 | 27.10 | Open sea | o8 |
| Samso | 10.673250 | 55.857717 | 14.7 | 27.60 | Open sea | o163 |
| Samso | 10.666917 | 55.852217 | 14.9 | 27.60 | Open sea | o164 |
| Samso | 10.256400 | 55.833950 | 15.0 | 27.60 | Open sea | o10 |
| Nordlige Lillebælt | 9.903067 | 55.239050 | 1.0 | 15.50 | Open sea | o124 |
