# Supplementary material for: Species Sorting of Benthic Invertebrates in a Salinity Gradient – Importance of Dispersal Limitation
Source: PLoS One. 2016 Dec 22;11(12):e0168908. doi: 10.1371/journal.pone.0168908 (PMC5179068; doi:10.1371/journal.pone.0168908)
Supplement: S4 File — (DOCX) [file pone.0168908.s004.docx]

Literature consulted when evaluating geographical distribution of planktotrophic species.

Glockzin M, Zettler ML. Spatial macrozoobenthic distribution patterns in relation to major environmental factors- A case study from the Pomeranian Bay (southern Baltic Sea). J Sea Res. 2008; 59: 144-161.

Gogina M, Zettler ML. Diversity and distribution of benthic macrofauna in the Baltic Sea Data inventory and its use for species distribution modelling and prediction. J Sea Res. 2010; 64: 313-321.

Gogina M, Glockzin M, Zettler ML. Distribution of benthic macrofaunal communities in the western Baltic Sea with regard to near-bottom environmental parameters. 1.Causal analysis. J Mar Sys. 2010; 79: 112-123.

Jensen AS, Spärck R. Bløddyr II, Saltvandsmuslinger. Danmarks Fauna. 1934; 40: 1-208. (in Danish)

Hartmann-Schröder G. Annelide, Borstenwürmer, Polychaeta. In: Die Tierwelt Deutschlands und der angrenzenden Meeresteile und nach ihrer Lebensweise. 1996; 58: Teil, 2. Auflage, Gustav Fischer Verlag, Jena, 648pp.

Zettler ML, Bönsch R, Gosselck F. (2000) Verbreitung des Makrozoobenthos in der Mecklenburger Bucht (südliche Ostsee) - rezent und im historischem Vergleich. Mar Sci Rep Inst Ostseeforsch Warnemünde. 2000; Germany, 144 pp.

Ziegelmeier E. Die Schnecken (Gastopoda Prosobranchia) der deutschen Meeresgebiete und brackigen Küstengewässer. Helgol Wiss Meeresunters. 1966; 13: 1-61.
