# Supplementary figures and images for: Species Sorting of Benthic Invertebrates in a Salinity Gradient – Importance of Dispersal Limitation
Source: PLoS One. 2016 Dec 22;11(12):e0168908. doi: 10.1371/journal.pone.0168908 (PMC5179068; doi:10.1371/journal.pone.0168908)

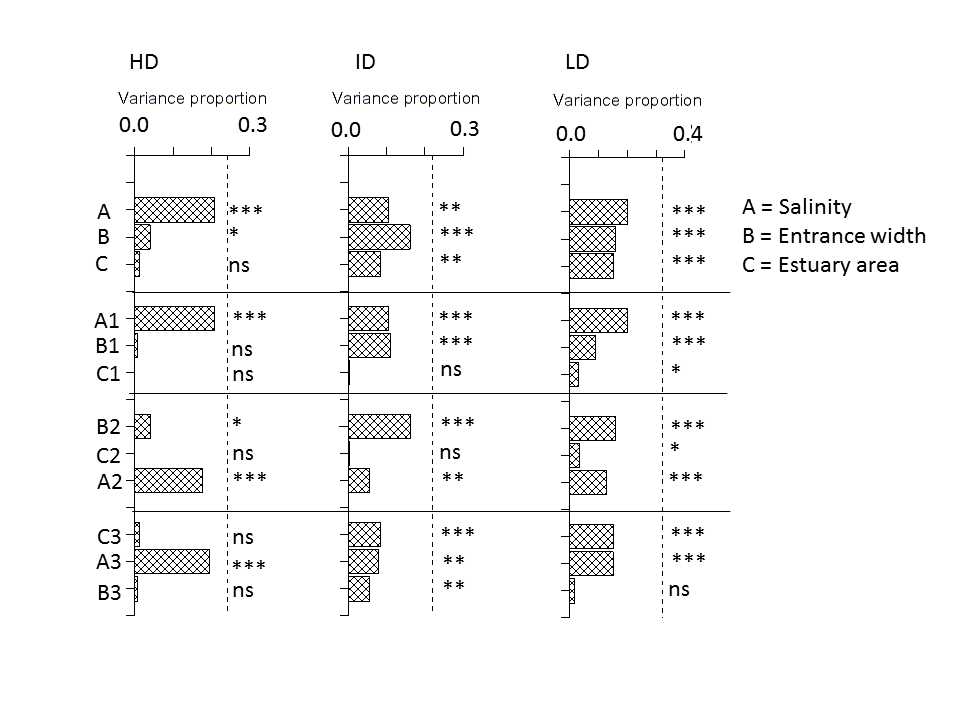

Supplement: S1 Fig — HD = High dispersive /planktotrophic species, ID = Intermediate dispersive /Lecithotrophic species and LD = Low dispersive / direct benthic development species group. Variance proportion = the proportion of total variance explained by the predictor (S2). Top sequence (A,B,C) shows results from marginal tests and the following three sequences (A1,B1,C1 and B2,C2,A2 and C3,A3,B3) show results from sequential tests, where A1, B2 and C3 are first fitted to data and B1,C1,C2,A2, A3, B3 give the remaining independent variance explained after the previous variable has been fitted. Dashed vertical line indicates the total variance proportion explained by the variables. *** = P = 0.001, ** = P <0.01, * = P <0.05 and ns = P >0.05. (TIF) [file pone.0168908.s005.tif]
